# Elevated local field potential power characterizes choreic storms identified by deep brain stimulator event capture

**DOI:** 10.3389/fneur.2026.1892893

**Published:** 2026-10-07

**Authors:** Pritha Bisarad, Kushala Shivaram, Elizabeth H. Le, Helen Magee, Sarah Hallgren, Lauren van der Werf, Melissa Duncan, Nicole Lucente, Francisco Ponce, Nicholas Schork, John A. Thompson, Michael C. Kruer

**Affiliations:** 1 Department of Child Health, University of Arizona College of Medicine—Phoenix, Phoenix, AZ, United States; 2 Departments of Cellular & Molecular Medicine, Neurology, and Translational Neuroscience and Program in Genetics, University of Arizona College of Medicine—Phoenix, Phoenix, AZ, United States; 3 Department of Rehabilitation Therapy, Phoenix Children’s, Phoenix, AZ, United States; 4 Functional & Stereotactic Neurosurgery Program, Department of Neurosurgery, Dignity Health Barrow Neurological Institute, Phoenix, AZ, United States; 5 Center for Translational Science, HonorHealth Research Institute, Scottsdale, AZ, United States; 6 Departments of Neurology & Neurosurgery, University of Colorado Anschutz, Aurora, CO, United States; 7 Pediatric Movement Disorders Program, Barrow Neurological Institute, Phoenix Children’s, Phoenix, AZ, United States; 8 Programs in Biomedical Informatics, Molecular & Cellular Biology, and Neuroscience, College of Health Solutions and School of Life Sciences, Arizona State University, Tempe, AZ, United States

**Keywords:** choreic storm, deep brain stimulation, local field potentials, neurogenetics, pediatric chorea

## Abstract

Some patients with hyperkinetic movement disorders may present with a severe and prolonged state of uncontrolled chorea and/or ballism, termed ‘choreic storm’, causing distress and prompting intervention. These events may be more frequent and severe in patients with childhood-onset movement disorders. Deep brain stimulation (DBS) can be effective for medically refractory dyskinesias including chorea, and the availability of event-based local field potential (LFP) sensing in clinically available devices present an opportunity to better understand the underlying neurophysiology of these episodic phenomena. We analyzed clinical, electrophysiological, and corroborating video data from a patient with a *de novo CEP85L* mutation (NM_001042475.2, c.2381A>G, p.Y794C) presenting with chorea-predominant dyskinetic cerebral palsy, microcephaly, and intellectual disability. The patient underwent bilateral pallidal DBS for medically refractory symptoms and showed improvement in her baseline chorea. However, choreic storms continued multiple times per week, requiring frequent rescue medications and/or emergency medical intervention. These episodes were associated with autonomic features and variable triggers. Family-initiated LFP event recording supplemented by concurrent video facilitated side-by-side analysis of clinical episodes, revealing elevated LFP power in the theta to low gamma frequencies during choreic storms compared to baseline event recordings. Episode-associated increases in LFP power captured during choreic storms may represent a putative within-patient electrophysiological correlate of these severe episodic exacerbations. Although requiring confirmation and extension in larger series, these observations suggest that adaptive stimulation paradigms could be useful for patients experiencing choreic storms.

## Introduction

Chorea define hyperkinetic movement disorders that are characterized by brief jumpy, jerky movements or movement fragments with an irregular pattern and distribution (1, 2). Clinically, chorea spans a phenotypic spectrum, ranging from subtle motor restlessness to dramatic, high amplitude, proximal movements with a flinging quality (i.e., ballism). These unwanted, abnormal movements can be exacerbated by sensation, passive or volitional movement, or stress (3).

In the United States, chorea prevalence is estimated on the order of 5–10 per 100,000 individuals, with variation across diagnostic, demographic and geographic groups (1). The etiology of pediatric and adult chorea is clinically heterogeneous and includes both acquired and genetic causes (4). Acquired etiologies encompass autoimmune, metabolic, vascular, infectious, and drug-induced conditions, some of which are potentially reversible (4, 5). In contrast, genetic choreas arise from mutations affecting neuronal signaling, synaptic function, or neurodevelopmental pathways, often resulting in chronic or progressive disease (4, 5). These diverse etiologies converge based on their impact on core motor circuitry (1). Although genetic choreas have been more rare, they are increasingly recognized, particularly in the pediatric population with advances in genomic diagnostics.

Chorea is most observed in Huntington Disease, followed by patients with dyskinetic cerebral palsy (CP). Dyskinetic CP is the second most common form of CP, representing nearly 14% of cases (6, 7). Mutations in genes such as *ADCY5*, *ATP1A3*, *FOXG1*, *SLC2A1*, *GNAO1*, *PDE10A*, and *NKX2-1* have been notably associated with chorea phenotypes in early-onset hyperkinetic movement disorders, including dyskinetic CP (6, 8–11). Patients with monogenic forms of chorea such as *ADCY5* and *GNAO1* have been reported to experience episodic worsening, with their chorea sometimes developing into violent storm-like motor phenotypes (5, 12–14).

Therapeutic interventions to treat choreic phenotypes include medications that are dopamine-depleting or D2 receptor-blocking, alpha2 adrenergic medications such as clonidine (15–18), as well as anticonvulsants such as carbamazepine, valproate (16, 18, 19) and benzodiazepines (16, 20). Additionally, surgical interventions like deep brain stimulation (DBS) have shown benefit in treating choreic symptoms in both pediatric and adult patients (20–23). Although DBS can be an effective tool in treating chorea, not all patients respond equally to the stimulation.

Local field potential (LFP) data collected from contemporary DBS devices could potentially serve as an informative electrophysiological markers, providing insights that may impact clinical management. Comparison of LFP profiles in Huntington’s-associated chorea showed that LFPs often demonstrated elevated power in high-beta to low-gamma frequencies (26–43 Hz), which correlated with severity (24). In this report, we present neurophysiological findings in an 8 year-old female with chorea-predominant dyskinetic CP associated with a *de novo CEP85L* (NM_001042475.2, c.2381A>G, p.Y794C) mutation, treated with bilateral pallidal DBS stimulation.

## Patient and methods

An 8 year-old girlwith a history of *CEP85L*-associated intellectual disability, microcephaly, and dyskinetic CP featuring chorea and ballism underwent bilateral globus pallidus pars interna (GPi) DBS placement along with Medtronic Percept RC at age 5. All data and videos presented here were collected after obtaining written informed consent from the patient’s family.

She was born at 39 weeks gestation (weight: 3.6 kg, height: 52 cm) via repeat cesarean section following a full-term gravida 2 para 2 pregnancy complicated by polyhydramnios. She was globally delayed in early milestone attainment, and concurrent microcephaly prompted neurological evaluation at 14 months of age. At that time, diffuse hypotonia accompanied by intermittent involuntary jerky movements was noted. Trio whole exome sequencing disclosed a heterozygous *de novo* Likely Pathogenic *CEP85L* variant (p.Y794C). She was diagnosed with dyskinetic cerebral palsy with predominant chorea at approximately 18 months of age (Figure 1). She underwent six electroencephalograms (EEGs), including long-term continuous video EEG monitoring, the first at age 16 months. None of these recordings identified epileptiform discharges but mild–moderate background slowing was consistently observed. Brain magnetic resonance imaging (MRI) disclosed anterior callosal dysgenesis, under rotation of the hippocampi, and periatrial T2 hyperintensity in addition to microcephaly. Since age 3, she experienced marked insomnia, with polysomnography (PSG) demonstrating poor sleep efficacy and obstructive sleep apnea noted at age 7.

**Figure 1:**
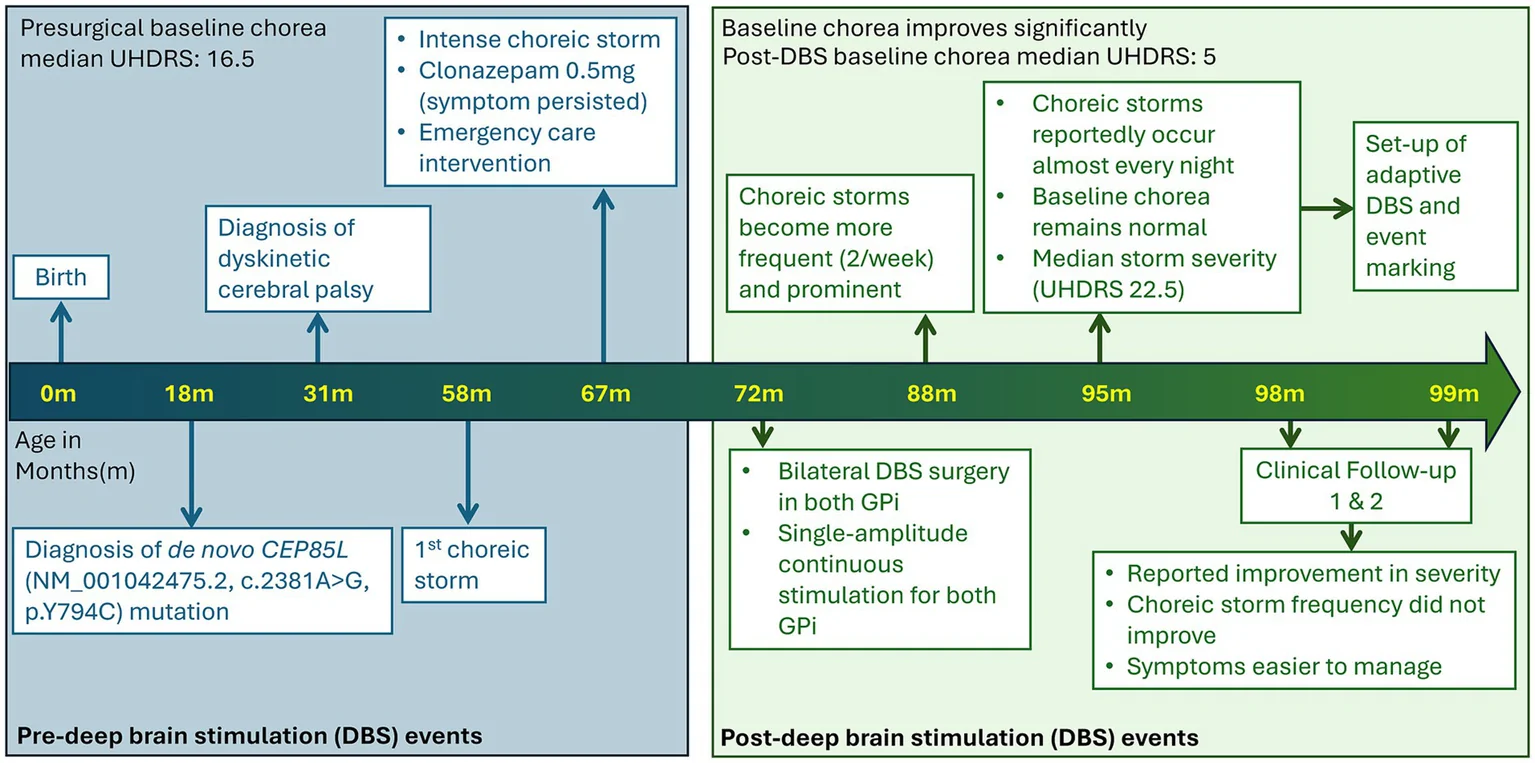
Timeline of major clinical events following birth, genomic characterization, and dyskinetic cerebral palsy diagnosis. Time of birth is considered time 0 (in months). All severity scores are reported as median Unified Huntington’s Disease Rating Scale (UHDRS) total chorea values.

Ballistic movements were noted in addition to chorea by her second birthday. These were occasionally forceful enough to cause painful impacts with nearby objects and/or people. Chorea severity was measured using the Unified Huntington’s Disease Rating Scale (UHDRS) total chorea subscale and median pre-DBS UHDRS ratings were 16.5. In addition to her baseline chorea, she also began to experience distressing periods of choreic storms (operationally defined as “a severe and prolonged state of uncontrolled chorea and/or ballism causing distress and prompting intervention”) that first required medication at age 4. These choreic storms were recurrent paroxysmal episodes of more dramatic choreic-ballistic movements that were distinctly more intense than the patient’s baseline chorea. These include involuntary, sometimes violent, large-amplitude flinging of the proximal extremities to the point of distress associated with sympathetic manifestations including tachycardia, hyperhidrosis and cutaneous flushing. These episodes often occurred in late afternoons or evenings; triggers include strong emotion and gastrointestinal distress. Mild to moderate events were primarily treated with clonazepam. During severe episodes, emergent interventions such as dexmedetomidine infusions were administered in our pediatric intensive care unit.

Given the severity and refractory nature of her dyskinesias, the patient was deemed a candidate for bilateral pallidal DBS treatment and underwent surgery at 5 years 11 month old (see Supplementary methods). Post-surgery, the neurostimulator was serially programmed to deliver continuous stimulation at fixed stimulation amplitude. During a later clinic visit, stimulation parameters were adjusted from continuous stimulation at a fixed amplitude to single-threshold adaptive DBS (aDBS) tracking physician-calibrated LFP frequencies of interest. Concurrently, user-reported event tracking was configured using the device’s BrainSense Events capability to identify the LFP features associated with choreic storms in this patient. Event tracking allowed the device to capture 30 s LFP snapshots based on family-initiated marking using the patient programmer (see Supplementary Table S2). Two physician-defined events were configured—‘Good Time’ to capture the patient’s mild baseline chorea, and ‘Choreic Storm’ to track choreic storms. The clinical team instructed the patient’s family to mark specific events at their onset. Bilateral LFP recording data was collected over a period of 17 weeks. LFP Snapshot power spectra (0–45 Hz, see Supplementary methods) stored on-device corresponding to each marked event were collected from corresponding JSON files. Retrieval of data from JSON files and subsequent data visualization and statistical analyses were carried out using Matlab (R2023b) scripts (Supplementary methods). Total number of recordings was calculated per event as power values were bilaterally averaged across hemisphere for each event. Between each event category, significance across canonical frequency bands was calculated using exact Mann–Whitney U test and multiple testing correction was performed using Benjamini-Hochberg statistics. Effect size was calculated using two statistically different approaches—Hodges-Lehmann method and Cliff’s delta method.

## Results

The patient demonstrated marked overall reduction in her chorea after DBS (median post-DBS UHDRS movement scale rating = 5; Figure 2). However, despite improvement in her baseline chorea (Table 1; Supplementary Video S1; Supplementary Table S1), choreic storms (Table 1; Supplementary Videos S2–S5; Supplementary Table S1) continued to occur frequently, almost daily at their peak, and continued to require periodic activation of emergency medical services, emergency department assessment, and intensive care unit admission.

**Figure 2:**
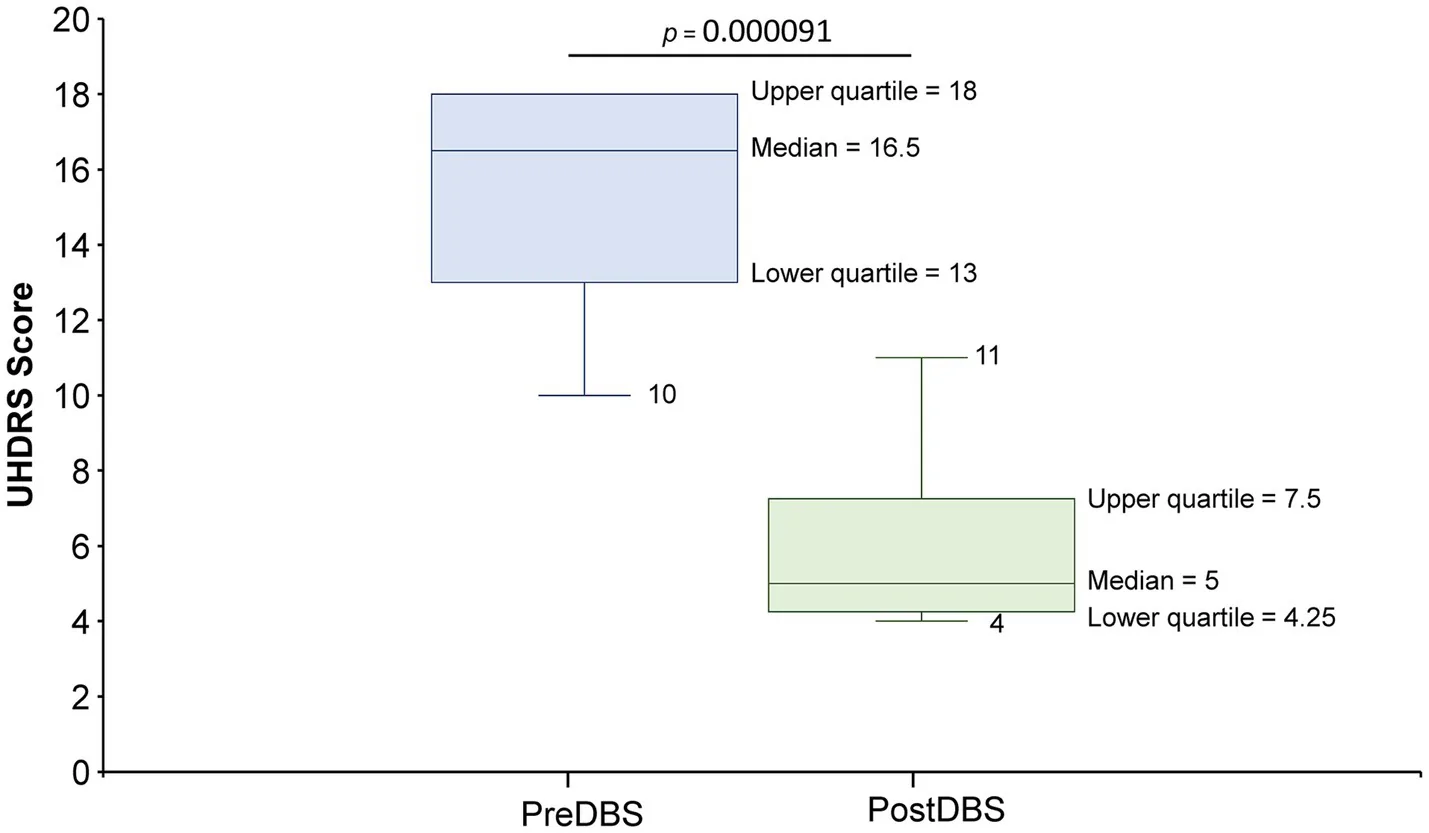
Comparison of pre- vs. post-DBS median UHDRS scores of the patient shows overall decrease in baseline chorea severity. Statistical significance was calculated using non-parametric Mann–Whitney U test.

**Table 1:** UHDRS scoring of chorea during baseline and choreic storm states from example videos.

Videos	Video S1		Video S2		Video S3		Video S4		Video S5	
States	Baseline chorea		Beginning of choreic storm		Choreic storm continues		Choreic storm + Clonazepam 1st dose (2 mg)		Choreic storm + Clonazepam 2nd dose (2 mg)	
Raters	R1	R2	R1	R2	R1	R2	R1	R2	R1	R2
Trunk	0	0	3	1	4	1	4	1	2	1
LUE	1	1	4	4	4	4	4	4	1	2
RUE	2	2	4	4	4	4	4	4	2	3
LLE	2	2	4	4	4	4	4	4	2	2
RLE	2	2	4	4	4	4	4	4	2	2
Face	0	0	1	0	2	0	2	1	1	0
BOL	1	1	3	2	3	3	3	2	1	2
Total UHDRS	8	8	23	19	25	20	25	20	11	12
Median UHDRS	8		21		22.5		22.5		11.5	

Green indicates baseline state; Orange indicates choreic storm state.

LUE: Left upper extremity; RUE: Right upper extremity; LLE: Left lower extremity; RLE: Right lower extremity; BOL: Bucco-oral-lingual; R1: Clinical Rater 1; R2: Clinical Rater 2.

Comparison of bilaterally averaged LFP snapshots from 3 ‘Good time’ events and 12 ‘Choreic Storm’ events show that the relative distribution of LFP power spectra remains the same during both event categories (Figure 3A). However, power spectral density (PSD) magnitudes during ‘Choreic Storm’ events are significantly higher than ‘Good Time’ events across specific LFP frequencies (Figure 3B), indicating increased LFP activity in multiple canonical frequency bands spanning from theta to low gamma. For sensitivity analyses, per event power values from left and right hemisphere individually were also investigated (Supplementary Figure S1) and show significant higher power in low beta band during choreic storm across both hemisphere. To further characterize the magnitude of this effect independent of statistical significance, we calculated the Hodges-Lehmann estimate of the median difference in power between ‘Choreic Storm’ and ‘Good Time’ events for each frequency band (Figure 3C). Positive estimates were observed across all bands, indicating consistently higher power during ‘Choreic Storm’ events, with the widest confidence intervals observed in the delta band, reflecting the smaller and more variable effect in this range in comparison to other bands. Cliff’s delta (|*δ*|), a standardized measure of effect size independent of the original power units, was similarly calculated for each band (Figure 3D); values ranged from small-to-medium in the delta band to large across the remaining bands, corroborating the pattern observed with the Hodges-Lehmann estimates and supporting a consistent, broadly distributed increase in LFP power during ‘Choreic Storm’ events.

**Figure 3:**
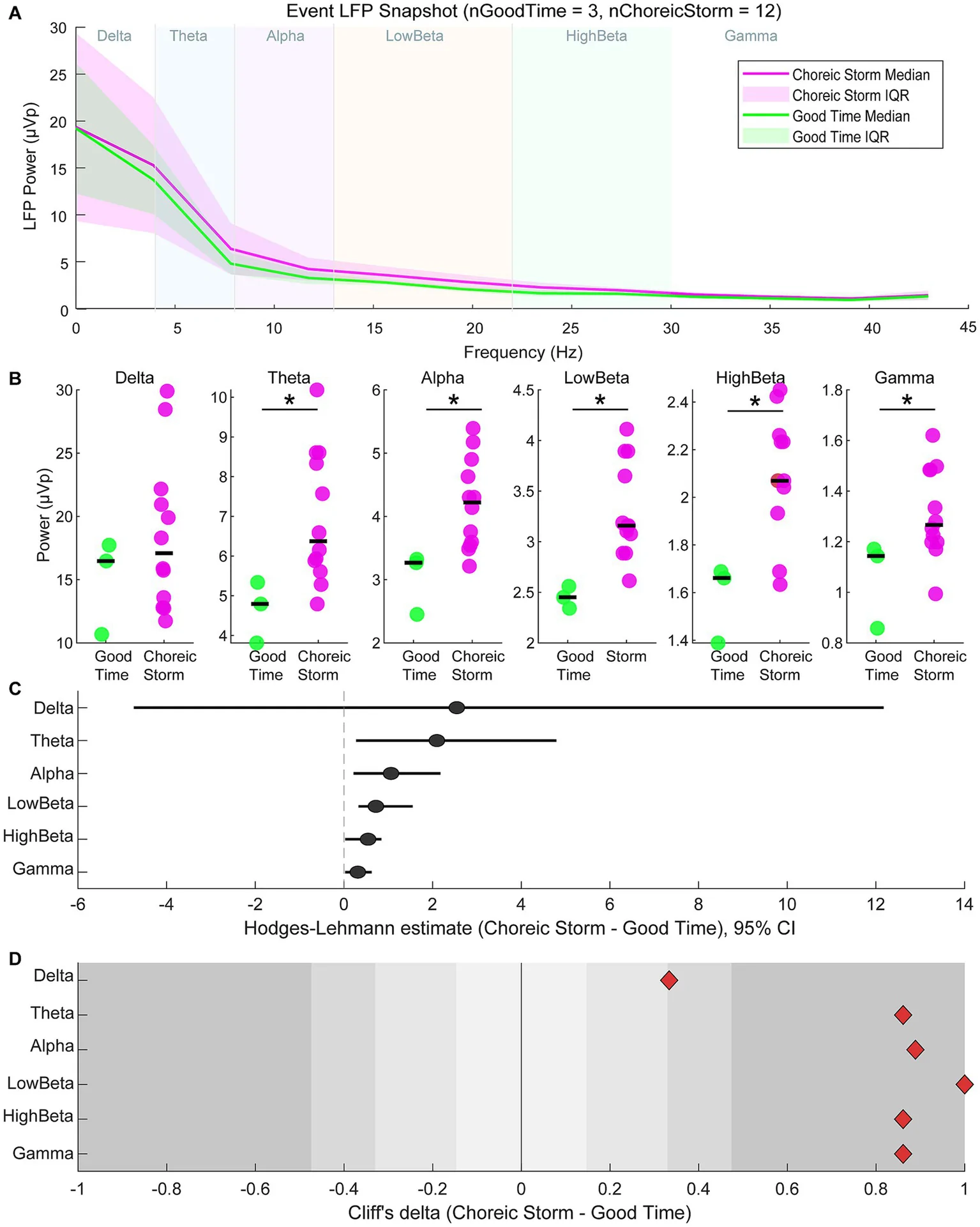
BrainSense Events snapshot shows significant differences in ‘Choreic Storm’ vs. ‘Good Time’ PSD magnitudes across different frequency features. **(A)** Median LFP power spectra (0–45 Hz) for Good Time (green, *n* = 3 events) and Choreic Storm (magenta, *n* = 12 events), with shaded regions indicating the interquartile range (IQR). Values are bilaterally averaged per event (left and right hemisphere power averaged prior to analysis) to avoid treating hemispheres as independent replicates. Canonical frequency bands—delta (0–4 Hz), theta (4–8 Hz), alpha (8–13 Hz), low beta (13–22 Hz), high beta (22–30 Hz), gamma (>30 Hz)—are indicated by background shading. **(B)** Event-level power values within each frequency band for Good Time (green, *n* = 3) and Choreic Storm (magenta, *n* = 12), with horizontal black lines indicating the group median. Each point represents one bilaterally averaged clinical event. * denotes statistical significance (*p* < 0.05) by exact Mann–Whitney U test, with *p*-values corrected for multiple comparisons across the six frequency bands using the Benjamini-Hochberg false discovery rate (FDR) procedure. **(C)** Hodges-Lehmann estimates of the median difference in power (Choreic Storm—Good Time) per frequency band, with 95% confidence intervals (horizontal lines). Positive values indicate greater power during Choreic Storm relative to Good Time. **(D)** Cliff’s delta per frequency band, quantifying the magnitude of the group difference on a scale from −1 to +1, using the Choreic Storm—Good Time convention (positive = Choreic Storm tends higher). Background shading indicates conventional effect-size interpretation bands: negligible (|*δ*| < 0.147), small (0.147 ≤ |*δ*| < 0.33), medium (0.33 ≤ |*δ*| < 0.474), and large (|*δ*| ≥ 0.474).

## Discussion

Here we present the case of an 8-year-old female patient with a *de novo CEP85L* mutation and recurring exacerbations of choreic movement, termed as choreic storms. Previously reported *CEP85L* mutations have been associated lissencephaly (OMIM #618873) with no prior reports of hyperkinetic movement-associated phenotypes (25–27). Although the clinical features described here may expand the recognized phenotypic spectrum associated with *CEP85L*, a comprehensive evaluation of the genotype–phenotype relationship is beyond the scope of the present report and will be addressed in ongoing work. This report intends to focus the discussion around the characterization of choreic storms.

In several ways, choreic storms may represent a form of ‘dyskinetic crisis’ (28, 29) similar to status dystonicus that many dystonia patients experience. We use the term ‘choreic storm’ to more precisely represent the nature and phenomenology of these events. Although treatment strategies for status dystonicus (30) and choreic storm may overlap in many cases, in other instances, appropriate treatments may differ, and precision is needed to optimally tailor therapy to a particular patient’s needs. Similar to other hyperkinetic spells, when choreic storms are prolonged, they can lead to acute and chronic complications including fatalities (28, 29). In addition, the recurrence and intensity of various forms of dyskinetic crises can lead to substantial physical and emotional burden on both patient and caregivers (29).

We leveraged the ability of a commercially available DBS device with chronic recording capability to capture events of interest during choreic storm states and to compare these recordings to epochs of stability. Analysis of family-identified event data indicates that choreic storms can be associated with elevated local field potential activity within the GPi, particularly in the theta and beta frequency bands, providing novel insight into their neurophysiology. This early observation is reminiscent of the increased beta band power recently described during episodes of status dystonicus (31). Our findings are also seemingly compatible with chorea-associated LFP findings from adults with Huntington’s chorea (23–25), particularly in regard to an association between symptom severity and higher LFP power in the beta and low gamma bands (23). However, in our index patient, the overall morphology of the captured LFP power spectra between these two states was relatively similar despite the differences in magnitude observed. This similarity in spectral morphology could potentially raise questions of what is driving the observed increase in power. Because choreic storm episodes involve substantially more chorea and ballism than good time periods, and because the recording platform captures only spectral power rather than the underlying waveform, some degree of movement-related signal contamination remains plausible, as the available data does not permit experimental verification of its presence or absence. Although the relatively uniform increase across frequency bands may reflect genuine neurophysiological change, the possibility of movement-related contamination cannot be exclusively negated and further validation studies are required to investigate potential movement related artifacts.

Though event tracking has been highly insightful in this case, it is worth note that event logging is wholly dependent on user (in this case, the family) participation. Events were not captured in an experimentally controlled environment; they were recorded in the patient’s home. This leads to some inherent uncertainty (i.e., estimation of time of onset for a particular event) but has potential trade-offs in generalizability (pragmatically, our findings suggest event capture has promise in a ‘real world’ deployment). It is important to note that because all recordings were obtained from a single participant and include repeated bilateral hemispheric measurements, the reported *p*-values should be interpreted judiciously as intra-subject observations, given that pseudo-replication cannot be entirely excluded. There is sparse literature regarding how pallido-pallidal interactions may impact interhemispheric LFP recordings in chorea, secondary findings suggest that there is low cross-hemispheric redundancy in frequency bands that are prominent in chorea with respect to GPi-DBS (24, 32). Given this uncertainty, and in the absence of more definitive evidence on the degree of hemispheric independence, we treated the clinical event rather than the hemisphere as the primary unit of analysis, to mitigate the effects of pseudo-replication, with effect size emphasized over *p*-values given the small sample. However, additional recordings in other patients will be needed to determine the ultimate utility of this approach.

We conclude that choreic storms likely represent under-recognized but important phenomenological events that can markedly impact patients and their families. Our data indicate that, similar to observations in status dystonicus, event capture paradigms may be valuable tools to identify changes in neurophysiology during this particular form of dyskinetic crisis. We provide proof-of-principle observations suggesting a putative electrophysiological correlate feature of choreic storm state, characterized by elevated LFP power across multiple canonical frequency bands, including theta and beta ranges. If confirmed, this suggests that adaptive stimulation paradigms may be of value in controlling these events.

## Data Availability

The original contributions presented in the study are included in the article/Supplementary material, further inquiries can be directed to the corresponding author.
