# Supplementary material for: Elevated local field potential power characterizes choreic storms identified by deep brain stimulator event capture
Source: Front Neurol. 2026 Oct 7;17:1892893. doi: 10.3389/fneur.2026.1892893 (PMC13682959; doi:10.3389/fneur.2026.1892893)
Supplement: Supplementary file 1 [file Supplementary_file_1.DOCX]

**SUPPLEMENTARY METHODS**

**Surgical and Programming Methods**

Deep brain stimulation (DBS) surgery was conducted out in this patient in November 2023. Bilateral globus pallidus pars interna (GPi)-targeted Medtronic (Dublin, Ireland) B33015 leads were placed, coupled to a Medtronic Percept™ PC neurostimulator with simultaneous LFP recording, streaming, and sensing capabilities. Surgeries were carried out without complications, and the patients tolerated the procedures well, returning to clinic for DBS activation approximately 2 weeks after combined lead and neurostimulator placement. Initial programming, post-surgery, was set-up to deliver fixed stimulation to both GPi based on separate thresholds. Adaptive DBS and BrainSense Event tracking was set up in October 2025, following 23 months after surgery.

**Supplementary Table 1. Descriptions accompanying supplementary videos.**

| Supplementary Video S1 | Video shows what patient's baseline chorea-ballism looks like when she is awake and comfortable. **UHDRS (chorea): 8** |
| --- | --- |
| Supplementary Video S2 | Video shows the start of a 'choreic storm'. Patient is exhibiting ballistic movements of all limbs and appears to be in distress. She has received her daily bedtime medications of quetiapine and clonidine to help her sleep. Mother marks an 'event' on patient programmer after the video. **UHDRS (chorea): 21** |
| Supplementary Video S3 | Video shows the continuation of the 'choreic storm'. Patient continues to have ballistic movements of all limbs to the point of distress. **UHDRS (chorea): 22.5** |
| Supplementary Video S4 | Video shows patient 5 minutes after first dose of 2 mg of clonazepam. Her choreic-ballistic movements have not reduced in response to medication. **UHDRS (chorea) : 22.5** |
| Supplementary Video S5 | Video shows patient 5 minutes after second dose of 2mg Clonazepam. Her choreic-ballistic movements appear to be markedly reduced. She appears drowsy and tired. **UHDRS (chorea): 11.5** |

**DBS Data Collection and Analysis**

LFP Snapshot through BrainSense Event was utilized in this case study. BrainSense Event allowed user-prompted capture of LFPs (30s) based on predetermined event labels defined by the programming clinician. It is important to note that the captured LFP undergoes immediate on-board Fast Fourier Transform (FFT) to convert it to power spectral density (PSD) in frequency domain, and the time-series data is not stored for future access (1). On device, FFT is applied over 1s windows with a frequency resolution of 0.98Hz. Also, LFP frequencies > 45Hz were censored on-device, due to low stimulation frequency (55Hz) (1), therefore only frequencies within 0-45Hz were considered for the analysis.

JSON files containing BrainSense Event data (LfpFrequencySnapshotEvents) were collected across 17 weeks and decoded in MATLAB (R2023b) using the loadJSON function from the Percept Toolbox (https://github.com/YohannThenaisie/PerceptToolbox). Events were categorized based on clinician-defined labels, “Storm” and “Good Time,” corresponding to choreic storms and baseline chorea, respectively.

LFP snapshot power spectra were extracted for each event. To avoid treating bilateral hemispheric recordings from the same clinical event as independent observations, left- and right-hemisphere power values were averaged to yield a single bilateral summary value per event and frequency bin, with the clinical event treated as the primary unit of analysis. A total of 3 "Good Time" events and 12 "Choreic Storm" events were analyzed at the event level. Custom MATLAB scripts were used for data visualization and statistical calculations. Median power spectral density (PSD) values and interquartile range were calculated within each event category and visualized. Discrete frequency values were binned into canonical frequency bands — delta (0–4 Hz), theta (4–8 Hz), alpha (8–13 Hz), low beta (13–22 Hz), high beta (22–30 Hz), and low gamma (30–45 Hz) — by averaging power across the frequency bins comprising each band, per event. Differences in bilaterally-averaged LFP power between event categories were assessed within each canonical band using the exact Mann-Whitney U test (equivalent to the exact Wilcoxon rank-sum test), which does not rely on a large-sample normal approximation and is therefore appropriate given the small and unbalanced group sizes. Resulting p-values were corrected for multiple comparisons across the six frequency bands using the Benjamini-Hochberg false discovery rate (FDR) procedure, and bands surviving correction were indicated with '*'. To characterize the magnitude of group differences independent of statistical significance, the Hodges-Lehmann estimate of the median difference (with 95% confidence interval) and Cliff's delta were calculated for each frequency band. As a sensitivity analysis, left- and right-hemisphere data were additionally examined separately, without bilateral averaging, to assess whether the direction of the observed effect was consistent across hemispheres.

**Supplementary Table 2. Date and time of LFP snapshot recordings (BrainsSense Event) for distinct event types based on caregiver-initiated input.**

| **Event Type** | **Event Recording Time** |
| --- | --- |
| Good Time | 10/24/2025 6:39pm |
| Good Time | 12/30/2025 6:52pm |
| Good Time | 01/16/2026 6:52pm |
| Choreic Storm | 01/06/2026 11:14pm |
| Choreic Storm | 01/08/2026 9:36pm |
| Choreic Storm | 01/09/2026 5:42pm |
| Choreic Storm | 01/13/2026 5:41am |
| Choreic Storm | 01/16/2026 11:30pm |
| Choreic Storm | 02/07/2026 10:10am |
| Choreic Storm | 02/10/2026 10:15am |
| Choreic Storm | 02/13/2026 10:43am |
| Choreic Storm | 02/14/2026 11:08pm |
| Choreic Storm | 02/17/2026 1:20am |
| Choreic Storm | 02/17/2026 1:31am |
| Choreic Storm | 02/17/2026 1:49am |

**Supplementary Figure 1: Hemisphere-level sensitivity: raw event-level data distribution (left vs right, Good Time vs Choreic Storm).** Event-level power values within each frequency band in left and right hemispheres for Good Time (green, n = 3) and Choreic Storm (magenta, n = 12), with horizontal black lines indicating the group median. Each point represents one clinical event per hemisphere. Statistical significance by exact Mann-Whitney U test is indicated in each case. * denotes p-values surviving multiple comparisons across the six frequency bands using the Benjamini-Hochberg false discovery rate (FDR) procedure.

**Supplementary References**

1.	Swinnen B, Buijink AWG, Stam MJ, Hubers D, de Neeling M, Keulen BJ, et al. Pitfalls and Practical Suggestions for Using Local Field Potential Recordings in Dbs Clinical Practice and Research. *J Neural Eng* (2025) 22(1). Epub 20250206. doi: 10.1088/1741-2552/adaeee.
